# Immediate Implant Placement and Provisionalization Using Natural Tooth Structure in the Anterior Esthetic Zone: A Case Report

**DOI:** 10.7759/cureus.104549

**Published:** 2026-03-02

**Authors:** Abdulaziz Y Alwaqyan

**Affiliations:** 1 Advanced Education in General Dentistry, Kuwait Oil Company (KOC) Hospital, Al Ahmadi, KWT

**Keywords:** anterior esthetic zone, dental implants, immediate implant placement, immediate restoration, implant provisionalization

## Abstract

Immediate implant placement with immediate restoration in the anterior esthetic zone presents a clinical challenge due to high esthetic demands and the need for precise surgical and prosthetic planning. When appropriate case selection and evidence-based protocols are followed, this approach may reduce treatment time, preserve peri-implant soft tissue architecture, and enhance patient satisfaction.

This case report describes the management of a compromised maxillary anterior tooth using immediate implant placement and immediate provisional restoration. Following atraumatic tooth extraction, an implant was placed in a prosthetically driven position, with adequate primary stability allowing for same-day provisionalization. The provisional restoration was designed to support soft tissue contours while avoiding functional loading during healing. Clinical and radiographic follow-up demonstrated favorable hard and soft tissue outcomes, with successful integration of the implant and satisfactory esthetic results. The patient reported high satisfaction with both function and appearance, highlighting a positive impact on quality of life.

This case emphasizes the importance of careful patient selection, meticulous surgical technique, and prosthetic planning when considering immediate implant placement and restoration in the anterior esthetic zone.

## Introduction

The anterior esthetic zone presents significant challenges in implant dentistry due to the high visibility of the region and the complex interplay between hard and soft tissues. Tooth loss in this area has a substantial impact on oral function, facial esthetics, and psychosocial well-being, making treatment planning particularly critical and outcome-sensitive [1,2].

Immediate implant placement with immediate restoration has been proposed as a treatment approach that may reduce overall treatment time, preserve alveolar bone architecture, and maintain peri-implant soft tissue contours when compared with conventional delayed protocols [3-5]. This protocol has gained increasing acceptance due to advancements in implant design, surgical techniques, and prosthetically driven planning concepts. Prosthetically driven planning integrates digital diagnostics and guided surgical workflows to optimize three-dimensional implant position relative to the planned restoration, thereby improving positional accuracy, primary stability, predictability, and esthetic outcomes [6,7]. In this context, “prosthetically driven position” refers to implant placement guided by the planned definitive restoration rather than solely by available bone anatomy, while “adequate primary stability” generally denotes sufficient insertion torque and/or implant stability quotient (ISQ) values to support immediate provisionalization.

However, immediate implant placement remains a technique-sensitive procedure and is associated with potential biological and esthetic risks if not carefully planned and executed [8,9]. Key determinants of success include socket integrity, achievement of primary implant stability, soft tissue biotype, accurate three-dimensional implant positioning, and careful prosthetic planning [10-12].

This case report presents the management of a compromised maxillary anterior region using immediate implant placement, followed by immediate provisional restoration. The report emphasizes clinical decision-making, integration of digital planning principles, surgical and prosthetic considerations, and short-term esthetic outcomes, with particular attention to patient-centered benefits, functional rehabilitation, and quality-of-life improvement.

## Case presentation

A 21-year-old patient presented with retained primary maxillary lateral incisors (#52 and #62). The patient’s chief concern was improvement of dental esthetics while maintaining a natural appearance. Clinical examination revealed retained primary teeth with acceptable periodontal health and stable soft tissues. Radiographic assessment included periapical radiographs, panoramic imaging, cephalometric analysis, and cone-beam computed tomography (CBCT), which confirmed the absence of permanent successors and demonstrated adequate bone volume and favorable anatomical conditions for implant placement.

**Figure 1:**
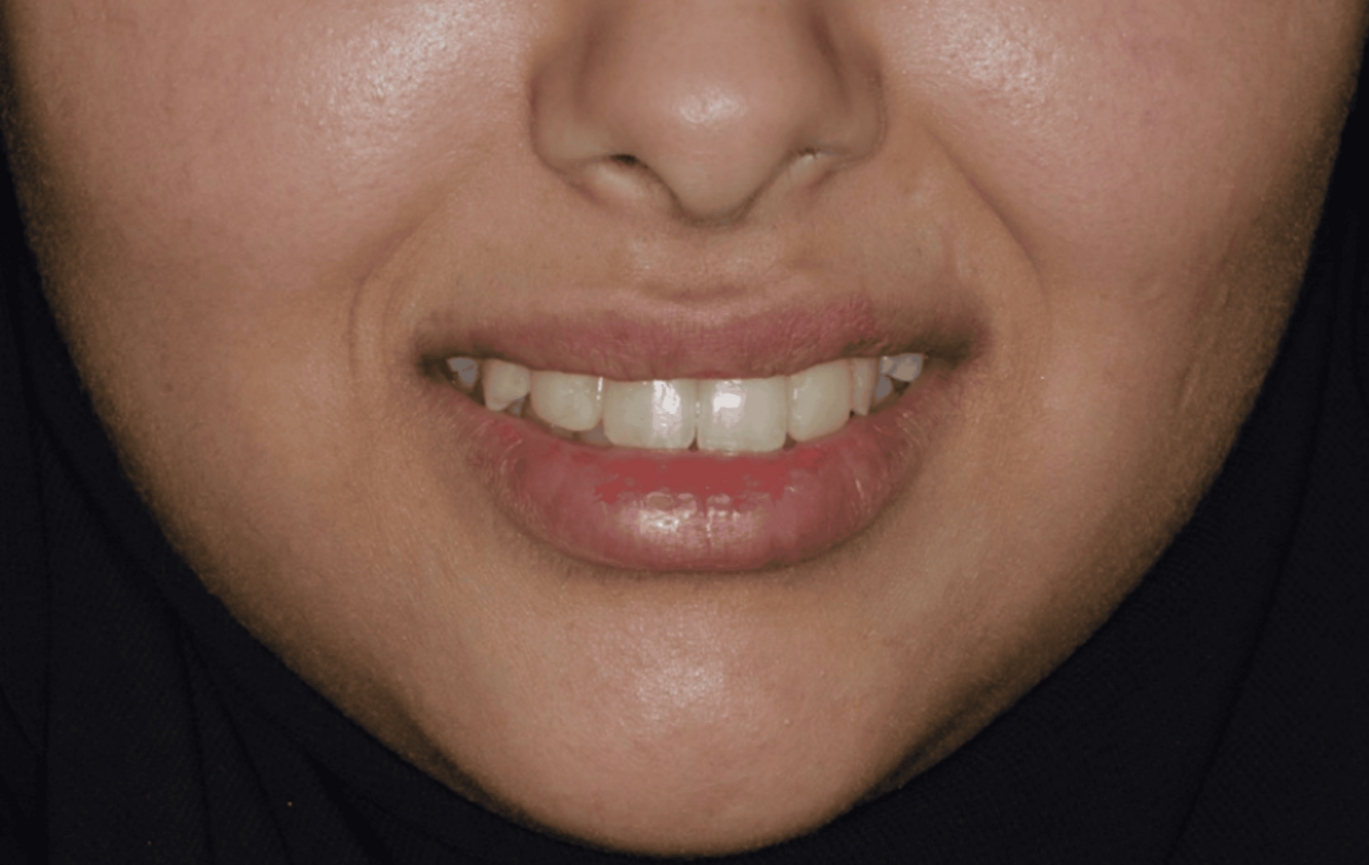
Preoperative frontal intraoral view showing retained primary maxillary lateral incisors Frontal intra-oral photograph demonstrating retained primary maxillary lateral incisors in a 21-year-old patient prior to implant treatment.

**Figure 2:**
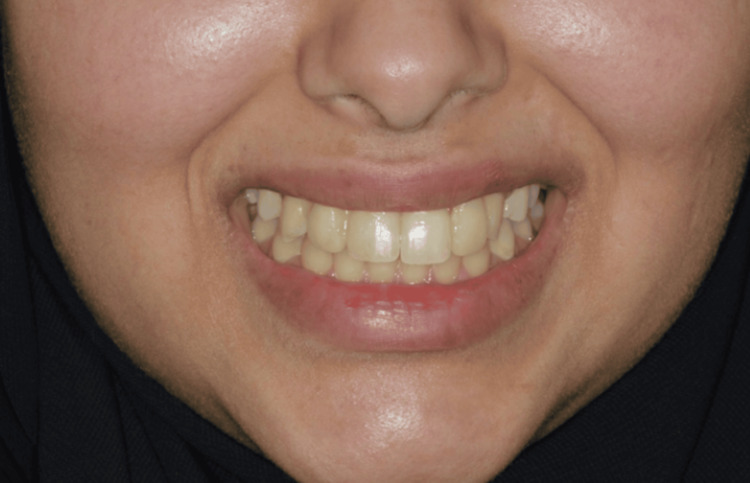
Preoperative smile view demonstrating esthetic concerns related to retained primary lateral incisors Smile photograph showing the esthetic impact of retained primary maxillary lateral incisors prior to treatment.

Extraoral and intraoral clinical evaluation demonstrated esthetic disharmony in the anterior region, with compromised smile esthetics and altered tooth proportions associated with the retained primary teeth (Figures 1-2). 

**Figure 3:**
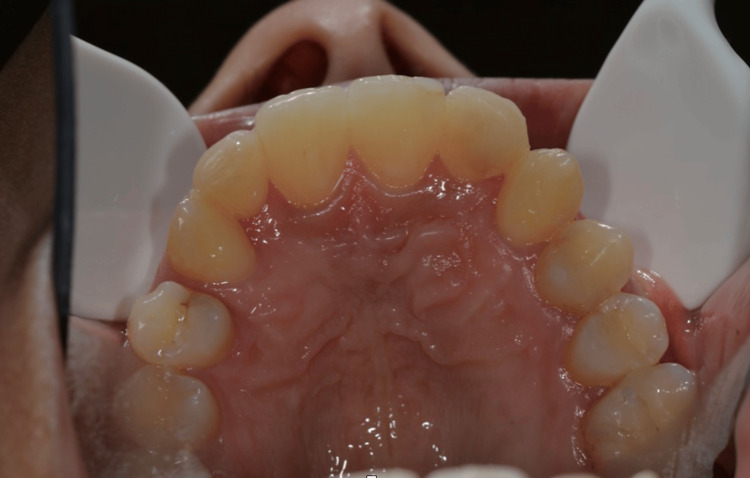
Preoperative maxillary occlusal view Occlusal view of the maxillary arch demonstrating retained primary lateral incisors and arch form prior to implant treatment.

Intraoral occlusal examination of the maxillary arch revealed retained primary lateral incisors, with favorable arch form and adequate space for implant-supported rehabilitation (Figure 3). 

**Figure 4:**
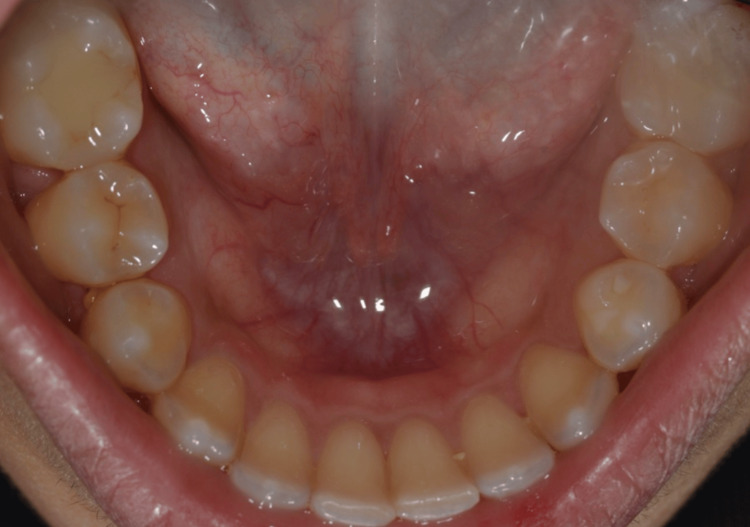
Preoperative mandibular occlusal view Occlusal view of the mandibular arch prior to implant treatment.

The mandibular arch demonstrated a stable occlusal relationship and healthy periodontal architecture (Figure 4). 

**Figure 5:**
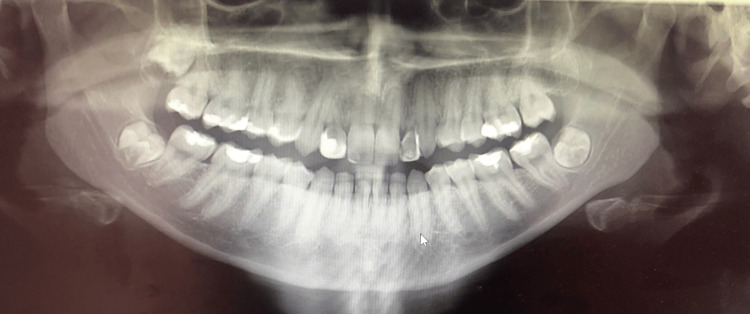
Radiograph - OPG OPG, Orthopantomogram

**Figure 6:**
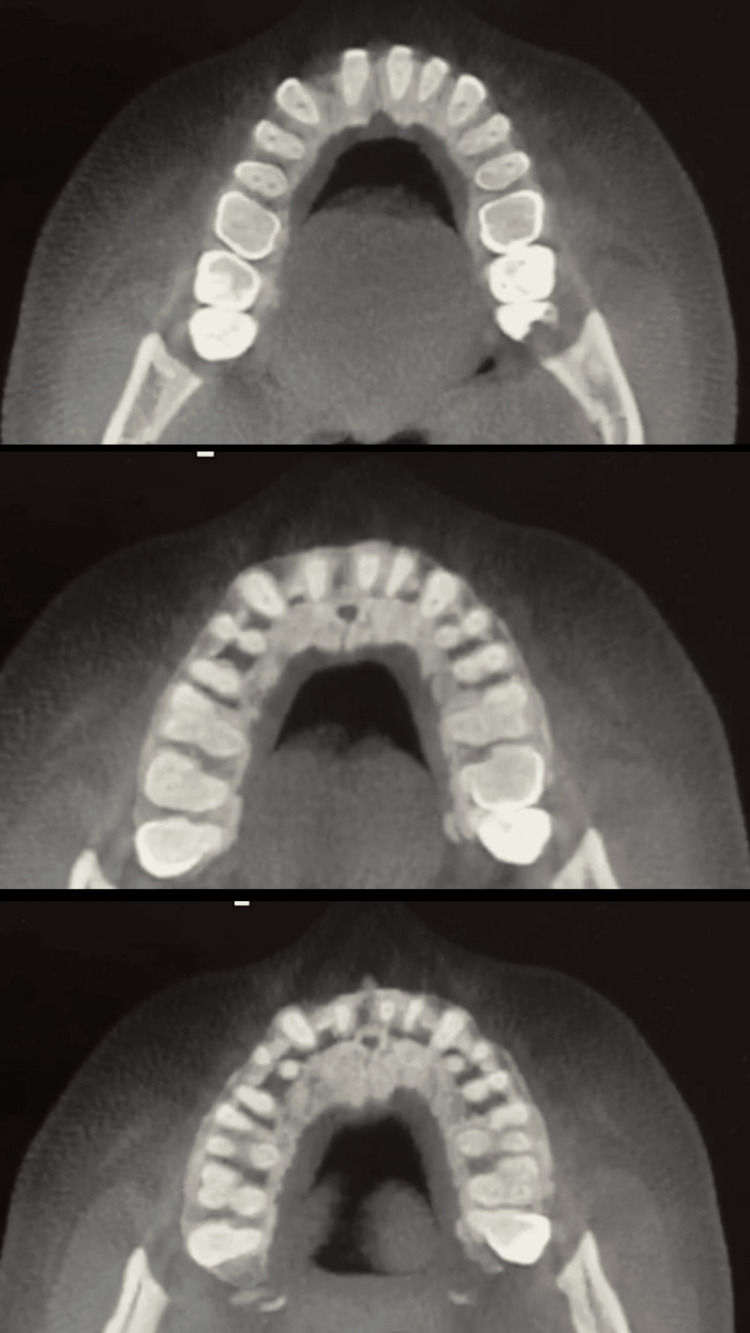
CBCT images show the bone density CBCT, cone-beam computed tomography

Panoramic radiographic evaluation confirmed the absence of permanent lateral incisors and adequate alveolar bone availability in the anterior maxilla (Figure 5). CBCT images show the bone density (Figure 6).

**Figure 7:**
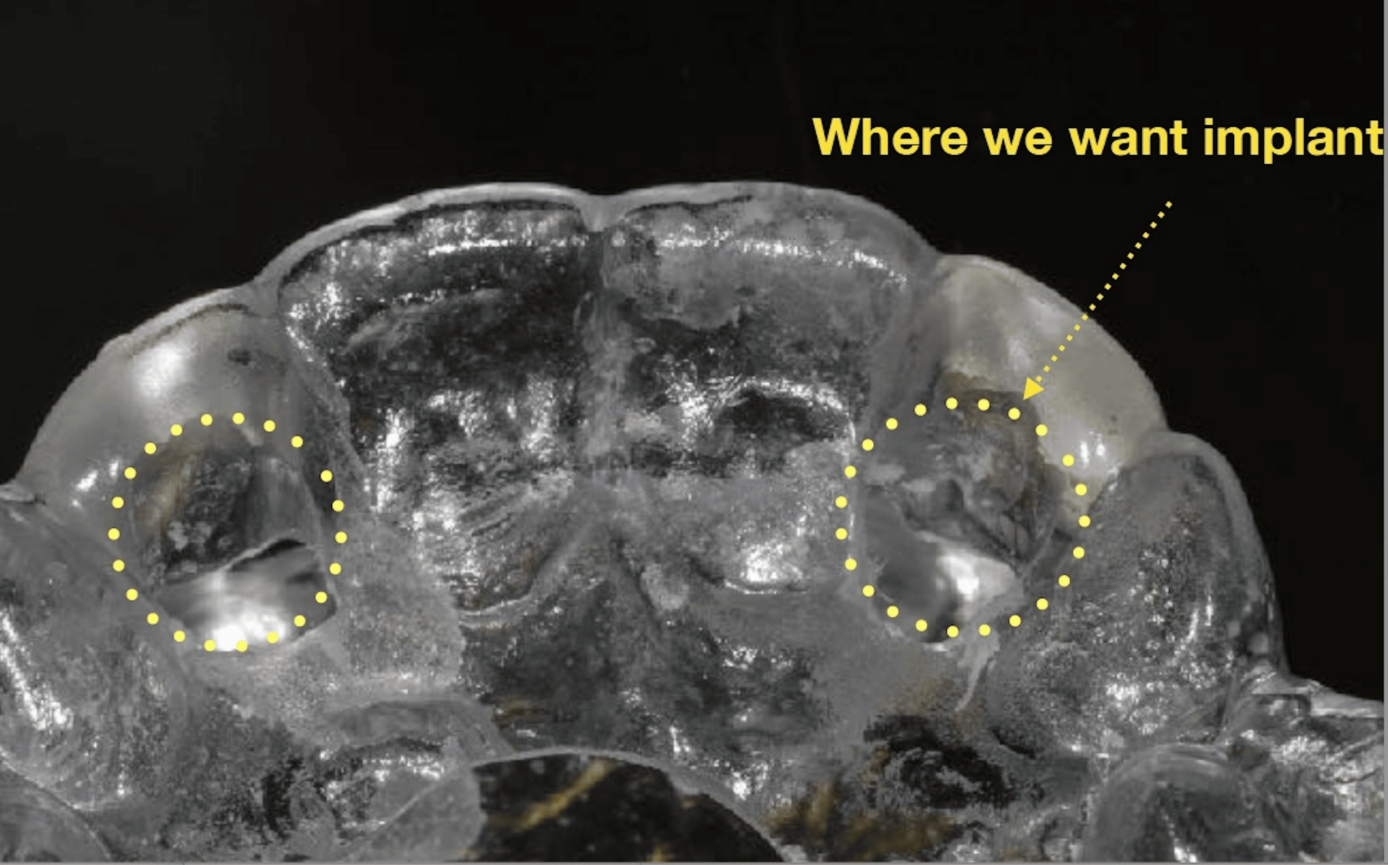
Surgical guide

Diagnostic impressions were obtained, and a poured study model was fabricated to aid in treatment planning. A comprehensive, prosthetically driven planning approach was adopted, integrating clinical findings, radiographic data, and model analysis. Based on this assessment, a surgical guide was designed to facilitate accurate three-dimensional implant positioning in the anterior esthetic zone (Figure 7). 

**Figure 8:**
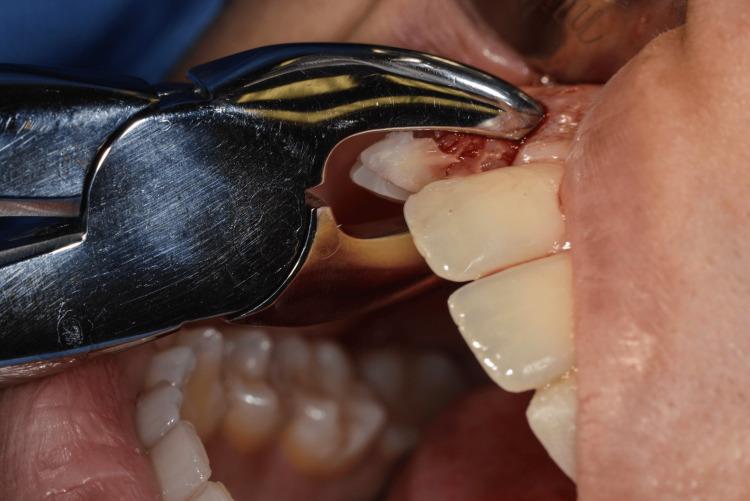
Extraction of primary laterals

**Figure 9:**
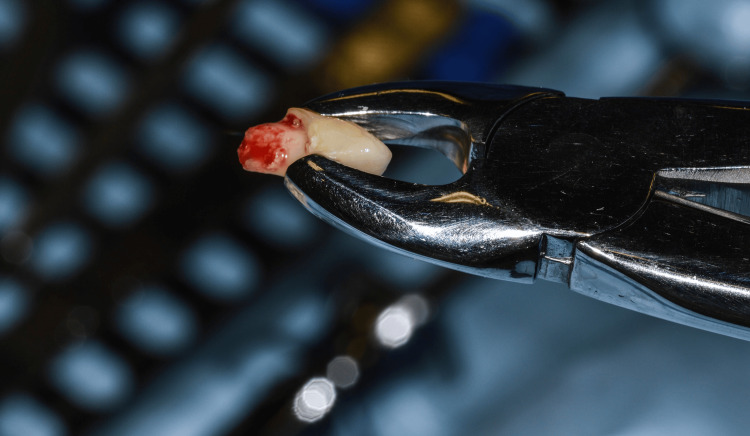
Primary lateral extracted

After discussion of treatment options with the patient, a treatment plan involving immediate implant placement with immediate provisional restoration was selected. Atraumatic extraction of the retained primary maxillary lateral incisors was performed, with careful preservation of the socket walls (Figures 8-9).

**Figure 10:**
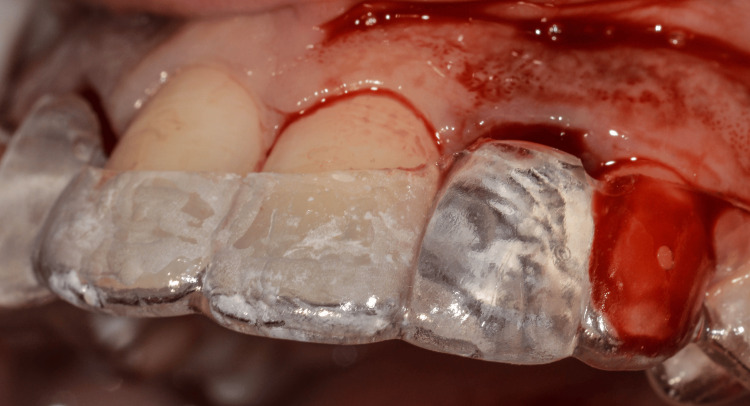
Surgical guide placed to ensure accuracy

**Figure 11:**
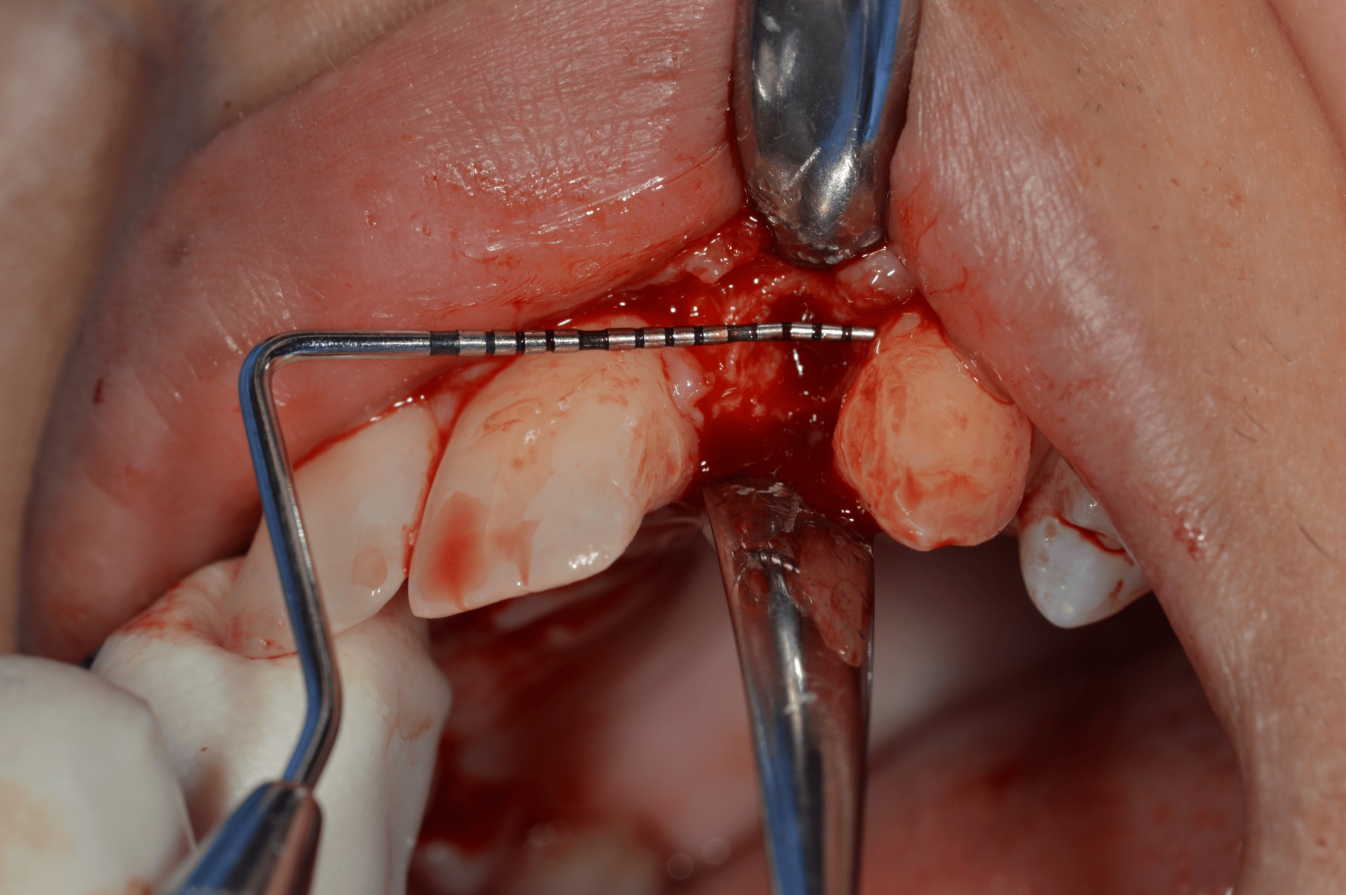
Measuring the socket width

**Figure 12:**
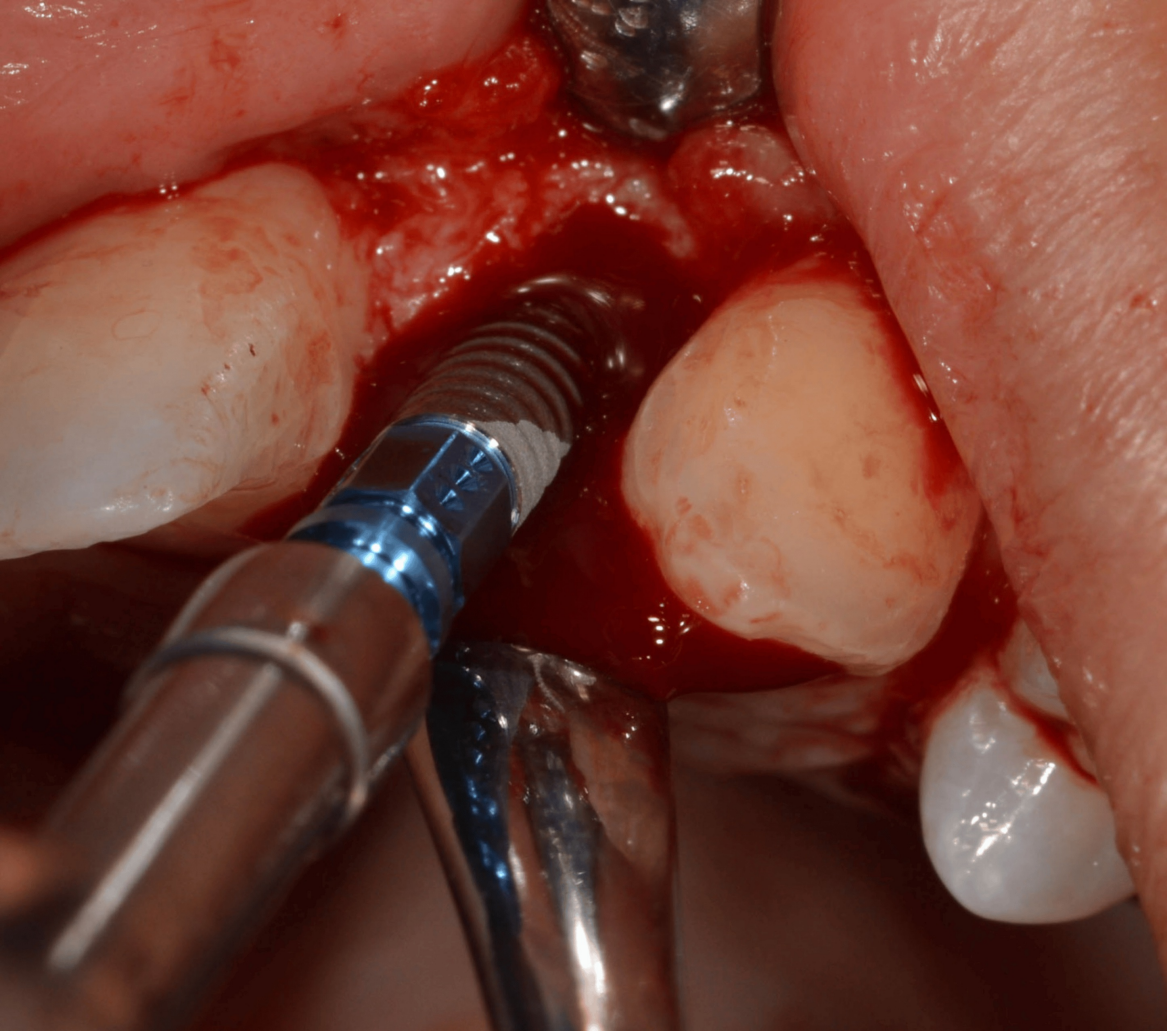
Implant placement

Guided implant placement was carried out using Straumann Bone Level Tapered (BLT) implants (2.9 mm diameter) to optimize implant angulation and depth, ensuring prosthetically driven positioning (Figures 10-12). 

**Figure 13:**
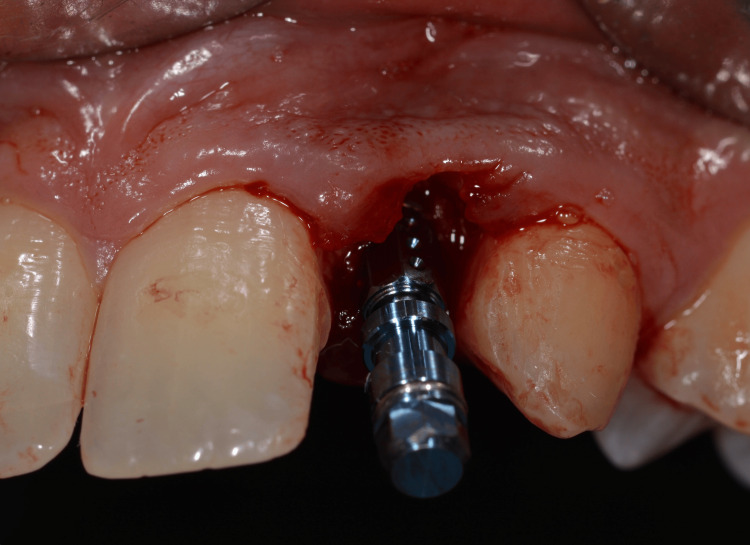
Implant placed

Primary implant stability of 35 Ncm was achieved for each implant, allowing for immediate provisionalization (Figure 13). 

**Figure 14:**
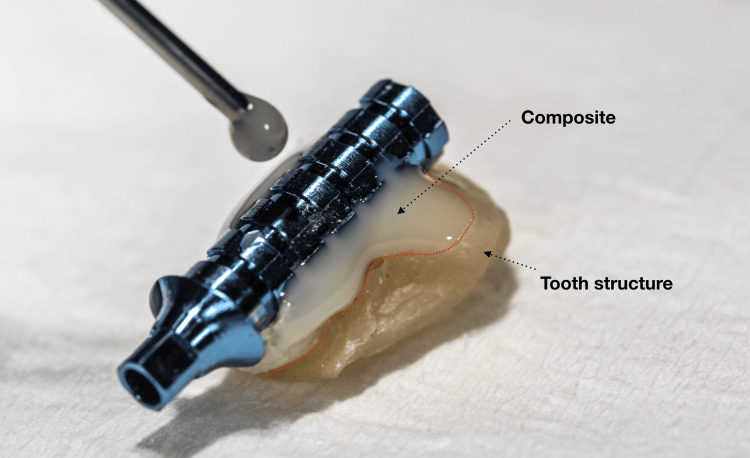
Natural tooth structure reuse

**Figure 15:**
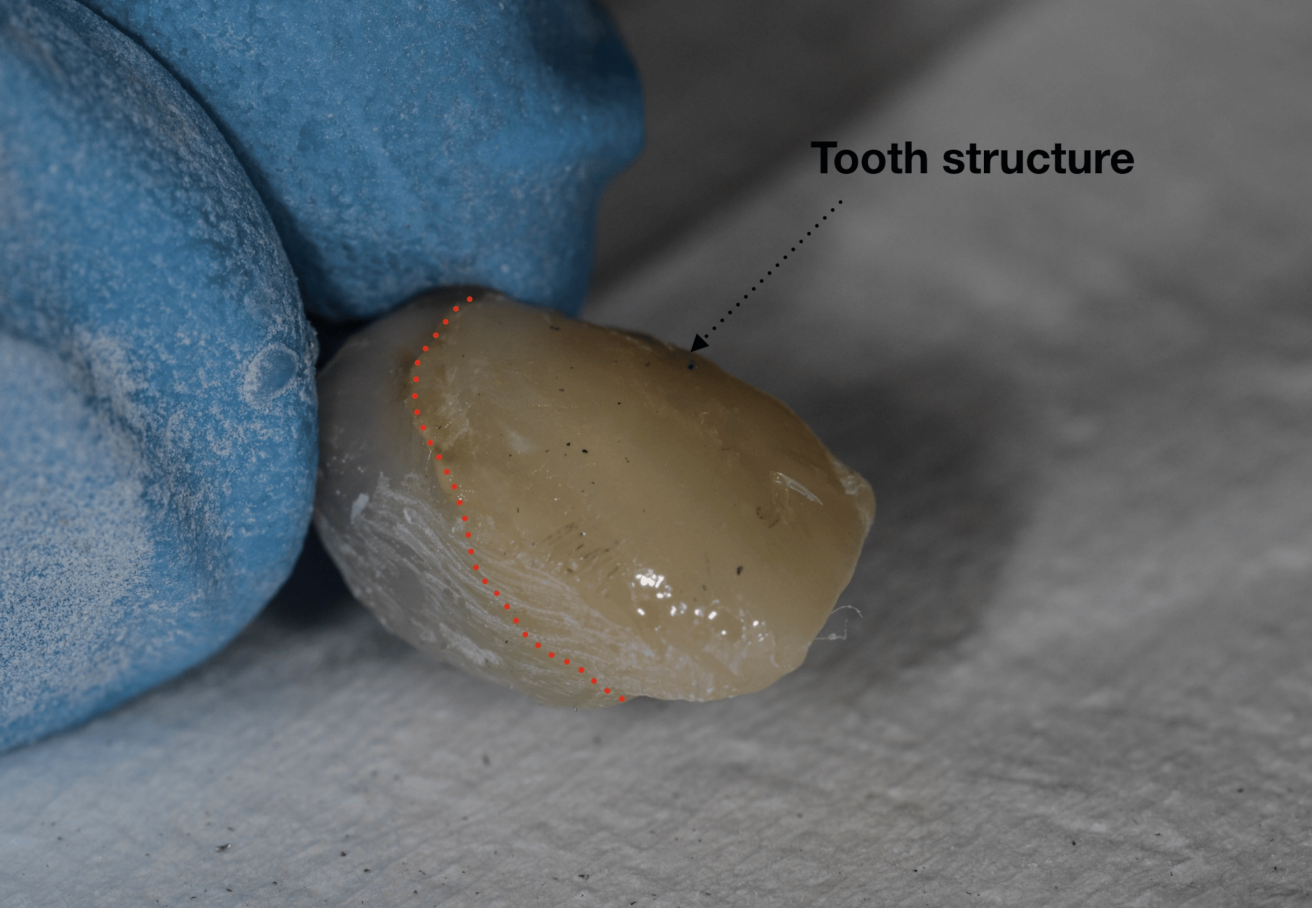
Immediate provisional restoration

**Figure 16:**
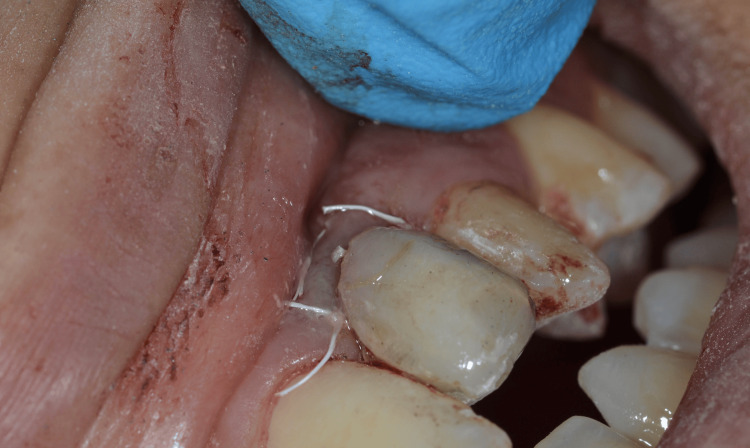
Immediate provisional restoration - intraoral

Following implant placement, screw-retained provisional restorations were fabricated on temporary abutments, with the abutment screws tightened to 15 Ncm in accordance with manufacturer recommendations. To enhance esthetic integration, a portion of the facial labial surface of the extracted natural tooth structure was incorporated into the provisional restoration and layered with composite resin. This technique was used to replicate the patient’s original tooth morphology, shade, and surface characteristics, thereby achieving a highly natural appearance (Figures 14-16).

**Figure 17:**
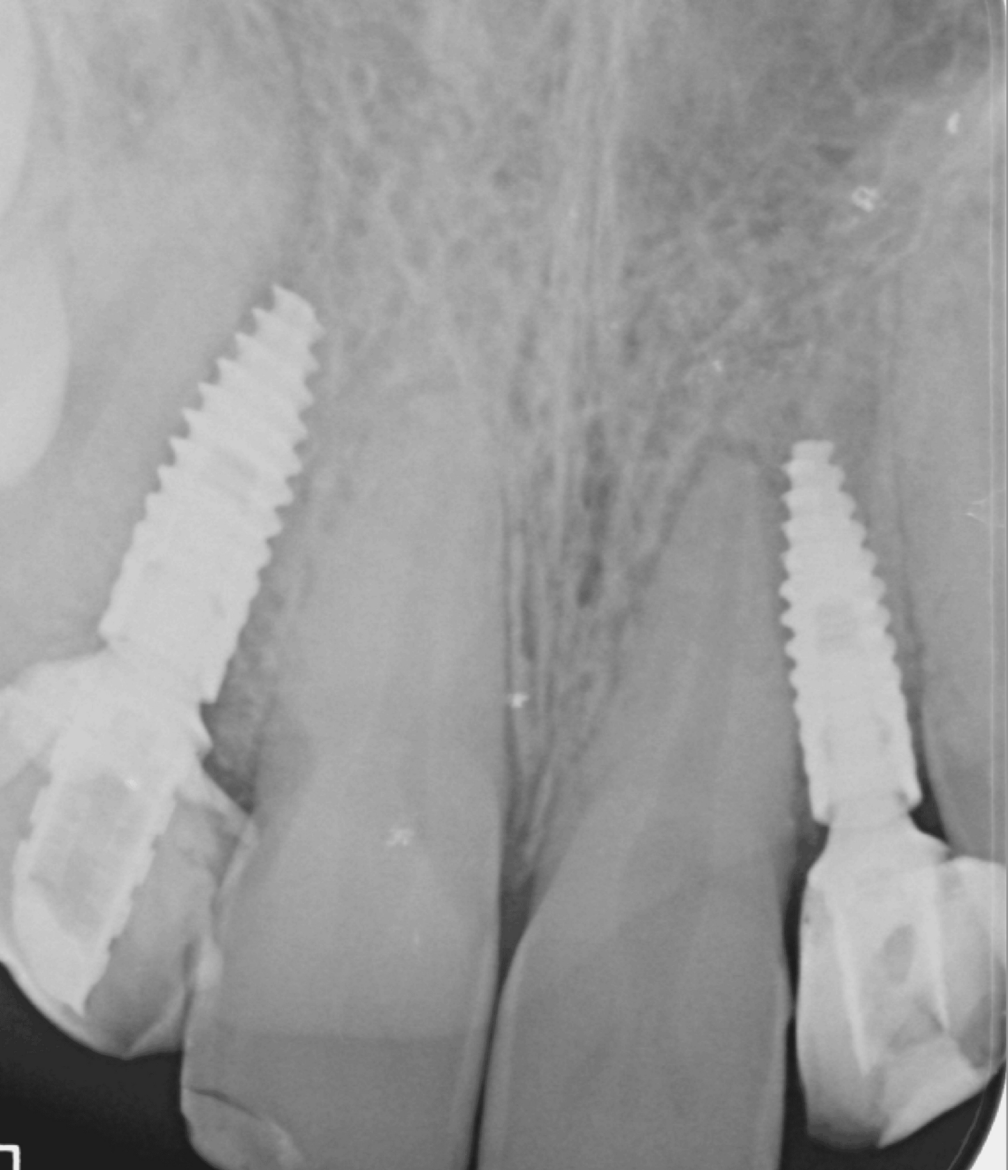
X-ray confirming the seating of the implant and the provisional crown

The immediate provisional restorations were delivered intraorally and adjusted to achieve proper emergence profile, cervical contouring, and soft tissue support. A periapical radiograph confirmed accurate implant positioning and appropriate seating of the provisional restorations (Figure 17).

**Figure 18:**
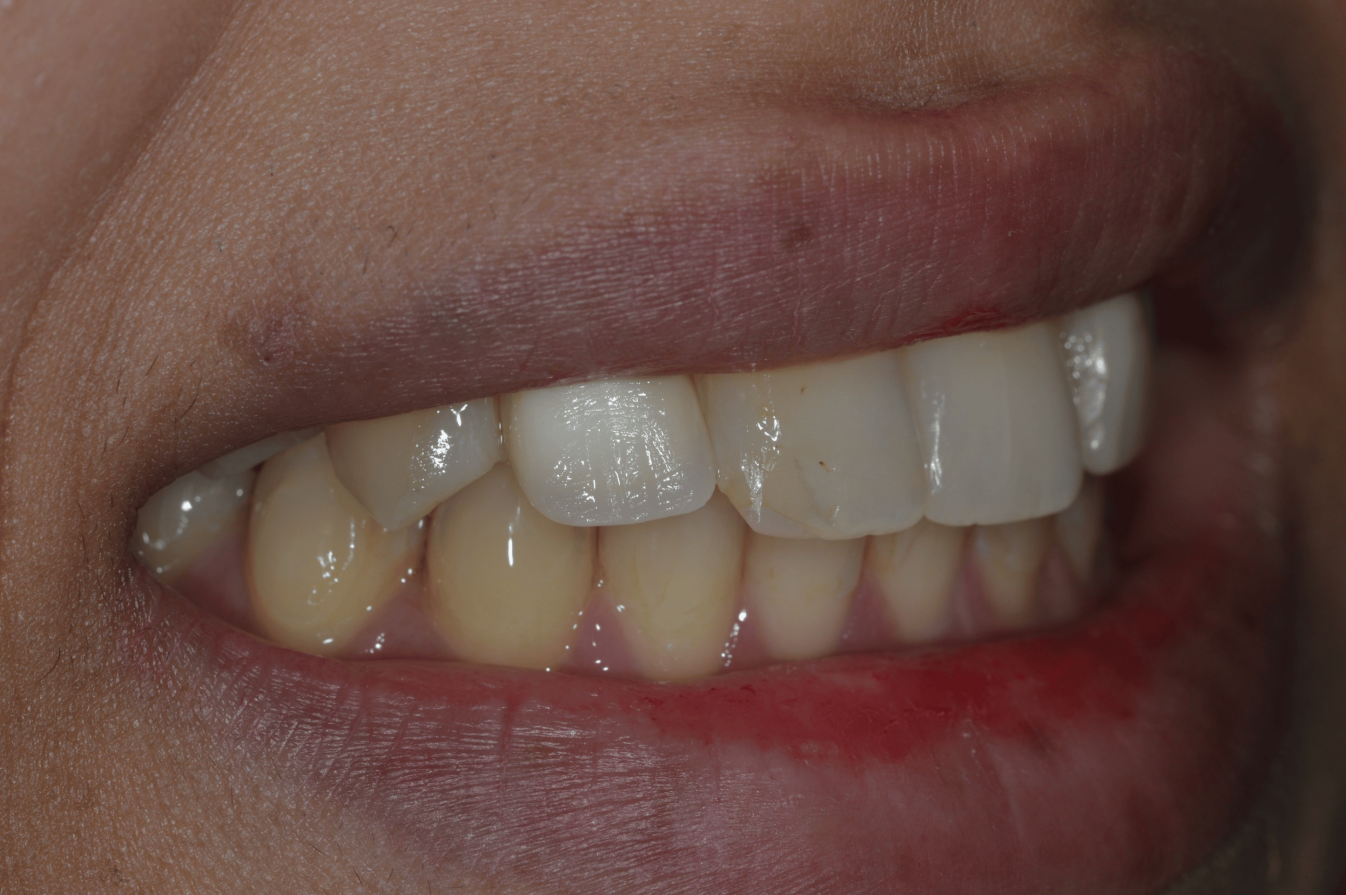
Postoperative smile view following immediate implant placement and provisionalization Smile photograph demonstrating favorable esthetic integration following immediate implant placement and provisional restoration.

**Figure 19:**
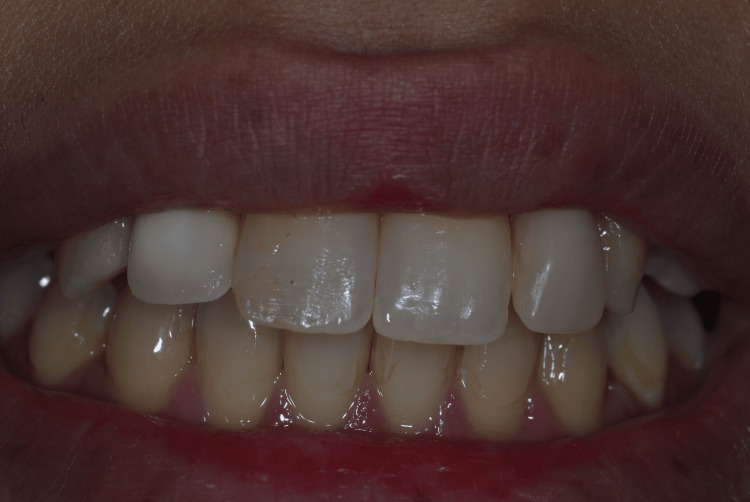
Postoperative smile view following immediate implant placement and provisionalization

The provisional restorations were adjusted to eliminate occlusal contacts in both centric and eccentric movements. Postoperative healing was uneventful, and peri-implant soft tissues demonstrated favorable contour and stability during follow-up. The patient reported high satisfaction with the esthetic outcome, particularly the natural appearance achieved through the use of autogenous tooth structure and immediate provisionalization. Postoperative smile evaluation demonstrated excellent esthetic integration and harmonious soft tissue architecture (Figures 18-19).

Follow-up was performed at one week, one month, three months, and six months. Peri-implant probing depths, bleeding on probing, soft-tissue condition, occlusal contacts, and periapical radiographs were recorded at each visit to evaluate implant stability and peri-implant tissue health [12]. The clinical and esthetic outcome assessment is provided in Table 1.

**Table 1:** Clinical and esthetic outcome assessment PES/WES, Pink Esthetic Score/White Esthetic Score

Parameter	Baseline	1 Week	1 Month	3 Months	6 Months
Implant insertion torque	35 Ncm	-	-	-	-
Implant stability (ISQ)	Not recorded	-	-	-	-
Peri-implant probing depth (mm)	-	Within normal limits	Stable	Stable	Stable
Bleeding on probing	No	No	No	No	No
Soft tissue condition	-	Favorable	Stable	Stable	Stable
Crestal bone level changes	-	Stable	Stable	Stable	Stable
Esthetic evaluation (PES/WES)	Not recorded	-	-	-	-
Patient satisfaction	-	High	High	High	High

## Discussion

Management of retained primary lateral incisors in young patients without permanent successors presents a complex clinical challenge, particularly in the anterior esthetic zone. In such cases, treatment success is determined not only by implant osseointegration, but also by the establishment of stable peri-implant soft tissues and an esthetic outcome that harmonizes with the patient’s smile [1,2].

Immediate implant placement with immediate provisionalization has been described as a viable treatment option in carefully selected cases, offering advantages such as reduced overall treatment time, preservation of alveolar bone architecture, and maintenance of peri-implant soft tissue contours [3-5]. However, this approach remains highly technique-sensitive. Predictable outcomes require meticulous case selection, atraumatic extraction, and precise three-dimensional implant positioning to minimize biological and esthetic complications [6,7].

In the present case, comprehensive preoperative assessment was central to clinical decision-making. A prosthetically driven treatment plan was adopted, and a surgical guide was utilized to facilitate controlled three-dimensional implant placement. In the anterior maxilla - where implant angulation, depth, and buccolingual positioning directly influence peri-implant tissue stability and esthetic outcomes - guided implant placement has been shown to improve positional accuracy and treatment predictability [8,9].

Immediate implant placement was selected due to intact socket walls and favorable bone conditions following atraumatic extraction. Achievement of adequate primary stability (35 Ncm) allowed immediate provisionalization, which supported peri-implant soft tissues and helped preserve pre-existing gingival contours during early healing. Previous studies have demonstrated that immediate provisional restorations can maintain peri-implant soft tissue architecture when occlusal loading is carefully controlled [10].

A distinctive feature of this case was the incorporation of the patient’s extracted natural tooth crown into the provisional restoration. This approach enabled accurate replication of natural morphology, surface texture, and shade, resulting in an immediate and highly natural esthetic outcome. The technique contributed to favorable soft tissue adaptation and high patient satisfaction at early follow-up. This concept aligns with biologically driven esthetic principles that emphasize preservation and replication of natural tissue characteristics in anterior implant therapy [11].

Despite the favorable short-term outcome observed, immediate implant placement with immediate provisionalization should be approached with caution. Long-term predictability depends on strict case selection, atraumatic surgical execution, sufficient primary stability, accurate prosthetic management, and diligent maintenance. Additionally, the reuse of the natural tooth crown is inherently case-dependent and requires intact tooth structure, favorable occlusion, and clinician expertise in anterior implant and esthetic rehabilitation.

It is important to acknowledge that conclusions drawn from a single case report are inherently limited. Validation through larger cohort studies and long-term follow-up investigations is necessary to confirm the reproducibility and stability of the described technique. Furthermore, the success of reusing the natural crown is highly dependent on specific clinical conditions and should not be generalized beyond carefully selected cases.

## Conclusions

Immediate implant placement with immediate provisionalization may represent a predictable treatment approach in the anterior esthetic zone when supported by careful case selection, comprehensive treatment planning, and precise surgical-prosthetic execution. In the present case, prosthetically guided surgery, achievement of adequate primary stability, and controlled occlusal loading contributed to favorable hard and soft tissue responses and a satisfactory esthetic outcome.

However, this technique should be reserved for carefully selected patients and performed by clinicians experienced in anterior implant therapy, as long-term success depends on meticulous planning, atraumatic surgical technique, and precise prosthetic management. Further studies with larger sample sizes and long-term follow-up are required to validate the reproducibility and clinical effectiveness of this approach.
